# Comparative Genomics of *Pinna rudis* and *Pinna nobilis* Reveals Conserved and Divergent Features of the Bivalve Defensome

**DOI:** 10.1002/ece3.73267

**Published:** 2026-03-25

**Authors:** Stéphane Coupé, Mathieu Foulquié, Maite Vázquez Luis, Elvira Alvarez Perez, Jean‐Marc Prévot, Nardo Vicente, Robert Bunet

**Affiliations:** ^1^ Université de Toulon, Aix Marseille Univ, CNRS, IRD, MIO Marseille France; ^2^ Institut océanographique Paul Ricard France; ^3^ Instituto Español de Oceanografía (IEO, CSIC), Centro Oceanográfico de Baleares. Muelle de Poniente s/n Spain; ^4^ Département Informatique Université de Toulon France; ^5^ Institut Méditerranéen de Biodiversité et Ecologie Marine et Continentale (IMBE), Aix‐Marseille Université, CNRS, IRD Avignon France

**Keywords:** annotated genomes, bivalve, critically endangered species, Fan mussel, immunity, MME, *pinna nobilis*, *Pinna rudis*, toll‐like receptors

## Abstract

The fan mussels *Pinna nobilis* and *Pinna rudis* have received increasing attention due to the mass mortality events (MMEs) that have severely impacted *P. nobilis* populations in the last decade. Strikingly, *P. rudis* was not affected by these events. Given that the two species can interbreed and that adaptive introgression has recently been suggested, it is essential to further investigate the genetic traits that may confer resistance to MMEs. This requires access to high‐quality, annotated genomes for both species. Here, we present the first high‐quality annotated genome of *P. rudis* , along with an updated genome assembly for *P. nobilis* . Comparative genomic analyses of gene sets associated with the “defensome” show that these genes are largely functionally conserved between the two species. Nevertheless, subtle differences in the number of annotations related to detoxification, stress response, and inflammation, along with variation in certain gene family expansions, suggest potential differences in susceptibility to environmental challenges. We also provide an updated characterization of the Toll‐like receptor (TLR) repertoire based on the classifications proposed for bivalves. Both species share a highly conserved set of TLRs, with most genes under purifying selection. Overall, these genomic resources represent a critical step forward for future research aimed at understanding the molecular mechanisms underlying disease resistance in *P. nobilis* and will ultimately support conservation efforts for this endangered species.

## Graphical abstract

We present the first high‐quality annotated genome of *Pinna rudis* and an updated genome assembly for Pinna nobilis, two Mediterranean fan mussel species differentially affected by mass mortality events. Comparative genomic analyses of gene sets associated with the “defensome” show that these genes are largely functionally conserved between the two species. Nevertheless, subtle differences in the number of annotations related to detoxification, stress response, and inflammation, along with variation in certain gene family expansions, suggest potential differences in susceptibility to environmental stressors, offering new insights into disease resistance and conservation of Pinna nobilis.

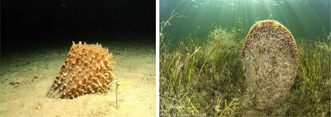

## 1 Introduction

**FIGURE 1:**
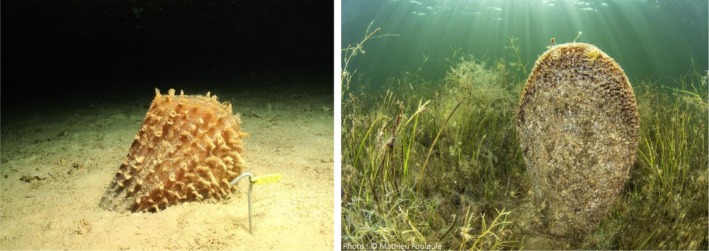
*Pinna rudis* (left) and *P. nobilis* (right) embedded in the sediment. Morphologically, *P. rudis* differs from *P. nobilis* by its tawny brown color and its large tubular, tile‐shaped scales, arranged along 5–10 radial ribs. It usually doesn't exceed a size of 30–40 cm, but can occasionally reach up to 50 cm.

*Pinna rudis* and *Pinna nobilis* are two closely related pen shell species belonging to the family Pinnidae (Figure 1). While *P. nobilis* is endemic to the Mediterranean Sea, *P. rudis* occurs both in the Mediterranean Sea, where it remains relatively rare and patchy distributed in the western part, being more scarce in the eastern part, but appears to be increasing in frequency and more commonly along the adjacent African Atlantic coasts (Gvozdenović et al. 2019; Zotou et al. 2023; Oprandi et al. 2024; Fassio et al. 2026; Kersting and Ballesteros 2021; García‐March and Kersting 2006; Nebot‐Colomer et al. 2016). These species differ notably in habitat preferences: *P. nobilis* typically inhabits seagrass meadows, especially *Posidonia oceanica* beds (Basso et al. 2015), while *P. rudis* is more often associated with rocky or coarse detrital substrates, and also in *Posidonia oceanic* installed on stony substrates.

Both species play key ecological roles. As filter feeders, they help reduce water turbidity and contribute to sediment stability. Their large shells serve as hard substrate for diverse epibiotic and endobiotic communities, providing refuge for a wide range of organisms (Basso et al. 2015; Trigos et al. 2014).

In recent years, *P. rudis* and *P. nobilis* have gained increasing attention due to the near collapse of *P. nobilis* populations across most of the Mediterranean sea and the posterior spread of *P. rudis* population (Zotou et al. 2023; Oprandi et al. 2024). Over the past decade, *P. nobilis* has experienced widespread mass mortality events (MMEs), primarily caused by infection with the protozoan parasite *Haplosporidium pinnae*, often in combination with bacterial and viral co‐infections (Künili et al. 2021; Lattos et al. 2020; Carella et al. 2023). As a result, the species has been classified as Critically Endangered by the IUCN (Kersting 2019). To date, only a few small populations of *P. nobilis* persist in the open sea, while the last healthy populations are confined to a limited number of coastal lagoons, although even these may not be exempt from ongoing MMEs (Labidi et al. 2023; Foulquie et al. 2023; Foulquié et al. 2024; Karadurmuş et al. 2024, 2025; Maresca et al. 2025). In contrast, *P. rudis* has not been affected by these mortality events and is currently regarded as a resistant species, as the parasite *H. pinnae* appears to have no detectable impact on it. Notably, *P. rudis* and *P. nobilis* are capable of interbreeding, and their hybrids have been observed to be resistant to MMEs (Vázquez‐Luis et al. 2021). This apparent resilience of *P. rudis* and *P. rudis* x *P. nobilis* hybrids raises important questions about the genetic and physiological mechanisms underlying resistance, particularly in relation to immune responses and host–parasite interactions.

We recently evidenced that some rare *P. nobilis* individuals surviving MMEs carried alleles of *P. rudis* origin, in immune‐related genes such as Toll‐like receptors (TLRs) (Coupé et al. 2023). This strongly points to the possibility that resistance traits may have been acquired through adaptive introgression from *P. rudis* .

Given the urgency of conserving *P. nobilis* and the unprecedented pace of species decline, identifying the specific genes of *P. rudis* origin that may confer resistance is of critical importance. Achieving this goal requires a comprehensive understanding of the gene repertoire in both species.

In this study, we present the first high‐quality genome assembly and annotation of *Pinna rudis* , along with a new genome assembly of *P. nobilis* , which updates the previously published shotgun genome (Bunet et al. 2021). This comparative genomic analysis enables us to explore gene family expansions and differences, with a particular focus on immunity‐ and detoxification‐related functions. We also use these genomic resources to update the repertoire of TLRs in both species, following the reference nomenclature proposed by Gerdol et al. (Gerdol et al. 2017).

## 2 Materials and Methods

### 2.1 Reference Genome Sequencing, Assemblies, and Annotations

Sampling of Pinna spp. was authorized by the relevant French and Spanish authorities. In France, authorization was granted by the Direction régionale de l'environnement, de l'aménagement et du logement d'Occitanie (Arrêté départemental n° 2023‐S‐8). In Spain, sampling permits included ESP 15/2023 for Pinna spp. in the Balearic Islands (2023) and SEN 376/23, issued by Cabrera National Park for sampling conducted within the park in 2023.

The DNA of *P. nobilis* and *P. rudis* was extracted from 1 mm (Oprandi et al. 2024) of mantle biopsies, using the DNAeasy Blood and Tissue kit (Qiagen). RNA was digested during the DNA extraction. DNA concentrations were determined using Qubit, and high‐molecular‐grade DNA was checked on a 0.8% agarose gel electrophoresis. PacBio libraries were prepared using the SMRTbell preparation kit following the manufacturer's instructions, then loaded into one 8 M SMRT cell and sequenced to generate circular consensus sequencing (CCS), also referred to as high‐fidelity (HiFi) reads. CCS output was quality trimmed using Fastp v0.23.4 (Chen et al. 2018) such that reads longer than 2000 bp and of quality score higher than 15 were kept. Long reads were analyzed using Kraken2 (Wood et al. 2019) (v25) to detect reads originating from bacterial contamination, with the confidence parameter set to 0.2, which represents a suitable compromise for long‐read data. Reads of likely bacterial origin were filtered out, and the resulting decontaminated fastq reads were then used for preliminary de‐novo assemblies performed using FlyE v2.9.3‐b1797 (Kolmogorov et al. 2019) with default settings.

To avoid excess false‐positive gene duplications due to high genetic divergence between two haplotypes, which may occur in eventually introgressed genomes, *P. nobilis* and *P. rudis* assemblies were processed using purge_dups (Guan et al. 2020).

In order to join broken contigs and reconstruct complete gene models, P_RNA_scaffolder (Zhu, Xiao, et al. 2018) was used using PE RNA‐seq reads available in NCBI, obtained from *P. nobilis* mantle tissue (SRR21820831) and haemocytes (SRR25473301), and from *P. rudis* tissue (ERR13847362), after reads filtering using Trimmomatic‐0.39 (Bolger et al. 2014) (LEADING:6 TRAILING:8 SLIDINGWINDOW:10:30 MINLEN:120) and kraken2 as described above, except that the ‐confidence parameter was set at 0.1. In order to assess whether both *P. nobilis* et *P. rudis* transcriptomes were complete, the fastq reads were assembled using Trinity (Grabherr et al. 2011) (Galaxy Version 2.15.1) (Galaxy Community 2024) and checked for completeness using BUSCO (Manni et al. 2021).

The completeness of both assemblies was assessed using BUSCO v. 6.0 with metazoa_odb12 and molluscan_odb12. Assembly statistics were obtained using QUAST v5.2.0. Repeat contents were assessed and classified, and soft‐masked using RepeatModeler (Flynn et al. 2020) and RepeatMasker v4.1.3 (Tarailo‐Graovac and Chen 2009) respectively. Open Reading Frames (ORF) were detected using BRAKER3 (Gabriel et al. 2024) available from Galaxy EU (https://usegalaxy.eu/) on unmasked assemblies. BRAKER3 was trained for gene prediction (GeneMark‐EX) using the above‐mentioned transcriptome paired‐end reads. Gene prediction results were assessed using TSEBRA (Gabriel et al. 2021) and for each gene, the longest predicted transcript was selected as the representative model. Predicted transcripts containing an identifiable CDS and longer than 90 nucleotides were translated into their corresponding proteins and functionally annotated using Diamond v2.1.11 (Buchfink et al. 2015) and either the NCBI non‐redundant (nr) protein database (April 2025 release), with the query‐cover and e‐value parameters set at 50% and 1e‐10, respectively, or a reference set of bivalve proteins (UniProt; Taxon ID 6544; November 2025 release), or RefSeq Protein, with the *e*‐value set at 1e‐5. A final consolidated annotation set was generated iteratively, starting from the annotations obtained with the molluscan dataset. For sequences initially annotated as “uncharacterized proteins,” the annotation was progressively refined by replacing them with matches from RefSeq Protein and, if necessary, from the nr database. Gene Ontology identifiers (GO id) were obtained using InterProScan 5 (interproscan‐5.74‐105.0‐64) (Jones et al. 2014).

### 2.2 Ortholog Identification and dN/dS Estimation

Orthologous transcript pairs between *Pinna nobilis* and *P. rudis* were identified using reciprocal best hit (RBH) analysis. Nucleotide sequences for each orthologous pair were retrieved from the corresponding transcriptome assemblies and translated into amino acid sequences using the standard genetic code. Protein sequences were aligned pairwise using MUSCLE (i.e., global mode) and filtered to remove low‐quality regions; poorly aligned columns and gap‐containing positions were excluded to minimize alignment artifacts and ensure compatibility with codon‐based analyses. Protein percent identity, aligned length, and alignment coverage were calculated for each filtered alignment.

To reduce potential biases, such as inflated interspecific divergence due to alternative exon predictions or truncated gene models, we examined the distribution of percent identity among syntenic orthologues in the closely related bivalves *Crassostrea gigas* and *C. angulata* , using MCScanX (Wang et al. 2012) with the following parameters: match score = 50; match size = 5, corresponding to the minimum number of colinear orthologs in a block; gap penalty = −1; overlap window = 5; *e*‐value = 1e–05 and max gaps = 25. The 75th percentile of these distributions corresponding to roughly 80% identity (Figure S1) was used as a cutoff, and only Pinna orthologues exceeding this threshold were retained. Accordingly, only orthologous pairs with a minimum protein identity of 80%, an aligned amino acid length ≥ 100 residues, and alignment coverage ≥ 70% of the shorter protein were included in downstream analyses.

Codon alignments were generated by mapping filtered protein alignments onto the corresponding nucleotide sequences using pal2nal. Rates of nonsynonymous (dN) and synonymous (dS) substitutions were estimated using codeml in the PAML package under a pairwise comparison model, assuming a single ω (dN/dS) ratio across sites and lineages. Codons containing gaps or ambiguous characters were excluded prior to analysis. The resulting dN, dS, and *ω* values were used for downstream statistical analyses, with ω interpreted as an indicator of selective pressure (*ω* < 1, purifying selection; *ω*≈1, neutral evolution; *ω* > 1, positive selection).

### 2.3 Genome Size Estimation and Genome Divergence Assessment

Genome size was estimated using a k‐mer‐based approach. For each species, 23‐mer frequency distributions were computed from the trimmed PacBio HiFi reads using Meryl (Rhie et al. 2020), and the resulting histograms were analyzed with GenomeScope v2.0 to infer genome size, proportion of unique sequences, and heterozygosity. To assess overall genome divergence, full assemblies and predicted coding sequences of both species were aligned using Minimap2 (preset ‐x asm20 or asm5) and analyzed for sequence similarity and structural differences.

### 2.4 Assessment of Gene Expansion Analysis

First, orthogroups were inferred from the predicted proteomes of *P. nobilis* and *P. rudis* using OrthoFinder v2 (Emms and Kelly 2019), which clusters genes into families based on sequence similarity and phylogenetic relationships. To improve the robustness of lineage‐specific inferences, we incorporated the NCBI RefSeq proteomes from *Dreissena polymorpha* (used as outgroup), *Mytilus galloprovincialis* , *Mytilus californianus* , *Mizuhopecten yessoensis*, *Atrina pectinata*, *Ostrea edulis* , *Saccostrea cucullata*, *Saccostrea echinata*, *Crassostrea virginica* , *Crassostrea gigas* , *Magallana angulata*, and *Magallana gigas*. Aligned orthologs were trimmed with trimAl (Capella‐Gutiérrez et al. 2009), and single‐copy orthologs from all species were concatenated for phylogenetic reconstruction using IQ‐TREE (Minh et al. 2020). Gene family expansions and contractions were then identified using CAFE5 (Mendes et al. 2020), which models gene gain and loss along a species phylogeny under a stochastic birth and death process. Following CAFE5 recommendations, we estimated the birth and death rate (λ) using a filtered set of orthogroups, excluding families with more than 100 genes, fewer than two genes, or those present in fewer than 50% of species. The estimated λ was then applied in a subsequent CAFE5 run on the complete dataset, assuming a uniform rate across the phylogeny. Gene families with FDR < 0.05 were considered significantly expanded. Results were summarized per species, and gene count tables were generated to compare changes between the two *Pinna* species. Expansions higher than two counts were considered.

### 2.5 Comparison of the Abundance of Genes Associated With Defense and Innate Immunity

To assess the similarity between the two species in their capacity to respond to environmental challenges, we quantified the number of annotated proteins related to immune system processes, stress responses, and detoxification, focusing on arbitrarily chosen gene annotation and Gene Ontology (GO) terms associated with these biological functions. Functional annotations obtained from InterProScan were used to validate the GO‐based classifications.

### 2.6 Toll‐Like Receptors (TLR) Description

TSEBRA‐based transcript selection improved overall annotation completeness; however, for gene families predominantly composed of single‐exon genes (such as Toll‐like receptors), TSEBRA‐derived models were often structurally inconsistent. This likely reflects the absence of splice‐junction evidence and limited RNA‐seq support, which are key signals used by TSEBRA for transcript ranking. For this reason, original BRAKER3 predictions were retained for the TLR repertoire analyses. Predicted TLR protein structures were identified using the SMART tool and subsequently classified following the criteria proposed for bivalves (Gerdol et al. 2017). Truncated TLRs, defined as sequences containing only TIR or LRR domains, were excluded from further analyses. Phylogenetic analyses were conducted based on the TIR domains since the evolution of TIR motifs reflects that of the extracellular LRR motifs (Saco et al. 2023). Protein sequences were aligned using the Clustal algorithm implemented in MEGA (Tamura et al. 2021) with default parameters. The best‐fit substitution model (LG + G) was determined using MEGA. A maximum likelihood phylogeny was then inferred with 1000 bootstrap replicates to assess branch support. The resulting tree was rooted using the TIR domain of a predicted TLR6 from the sponge *Amphimedon queenslandica* (XP_003383414.2). The repertoire of *P. nobilis* TLRs was refined by replacing three incomplete coding sequences with their previously described homologs and supplemented with one additional TLR previously reported in the species (Coupé et al. 2023).

## 3 Results and Discussion

### 3.1 Genome Assemblies and Annotation Statistics

Two annotated, high‐quality genome assemblies were generated for *P. nobilis* and *P. rudis* , with total sizes of approximately 810 and 760 Mb, respectively. For *P. nobilis* , a genome size of approximately 780 Mb was estimated by *k*‐mer analysis, with 65.2% of the genome identified as unique sequence and a heterozygosity rate of 1.06% inferred. For *P. rudis* , the *k*‐mer analysis yielded a smaller estimated genome size of approximately 650 Mb, with 68.6% unique sequence content and a heterozygosity rate of 2.23%. The discrepancy between the observed assembly sizes and the *k*‐mer based estimates, particularly in *P. rudis* , is likely explained by the assumptions of uniform sequencing coverage inherent to *k*‐mer methods, which may not hold in regions characterized by variable coverage or enriched in repetitive elements. In addition, heterozygosity is not expected to be uniformly distributed across the genome, and the presence of regions of hyper‐variability between haplotypes, as well as structural variants, may have resulted in the inclusion of a few megabases of sequence not shared between the two haplotypes. This type of “assembly inflation” is common whenever haplotype‐aware assembly methods or Hi‐C scaffolding are not used, as in the present study. Such discrepancies are consistent with many other bivalve genome projects, which also report larger assemblies relative to k‐mer–predicted genome sizes. Additional support for this interpretation was provided by a separate genome size estimate for another *P. rudis* individual obtained from resequencing using Illumina short reads at 10 × depth, which predicted a genome size of approximately 734 Mb (*personal data* (Coupé et al. 2023)).

The *P. nobilis* genome assembly consisted of 4263 contigs, with an L_50_ of 568 and an L_90_ of 1946 (Figure S2). Genome completeness, assessed using BUSCO with the eukaryota_odb12 and mollusca_odb12 datasets, was greater than 95%, with fewer than 1.2% of genes identified as duplicated. Gene prediction identified 47,593 protein‐coding sequences, of which 33,053 (69.4%) were annotated, similar to previous estimation (Bunet et al. 2021). The *P. rudis* genome assembly comprised 3422 contigs, with an L_50_ of 359 and an L_90_ of 1349. BUSCO analysis similarly indicated more than 93% completeness, with less than 1.7% of genes duplicated (Table 1; Table S1). A total of 40,088 protein‐coding genes were predicted, 29,146 (72.7%) of which were successfully annotated. Filtering out contigs shorter than 30 kb had minimal impact on overall genome completeness or the number of annotated proteins, resulting in only a roughly 2% reduction in total genome size. Correspondingly, L_50_ and L_90_ values decreased slightly (Table S1).

These gene counts are within the range observed in other molluscs and the high percentage of annotated genes supports the biological relevance of the predicted proteomes (Zhang et al. 2012; Wang et al. 2017; Gundappa et al. 2022; Ford et al. 2025). Also, the negligible impact of contig filtering suggests that assemblies are not heavily fragmented in functionally important regions, reinforcing the quality and usability of these genome assemblies. BUSCO run on predicted CDS also shows 93% of completeness, slightly lower than completeness obtained from genome assemblies (Table S1). Moreover, the similarity in length metrics further suggests that both genomes likely encode full‐length and biologically functional proteins.

Most annotated proteins exhibited top BLAST hits to sequences derived from bivalve species (Figure S3). As similarly observed in other mollusc genomes and predicted transcriptomes, a notable proportion of predicted coding sequences lacked functional annotation or were classified as uncharacterized or hypothetical proteins (approximately 26% and 28% for *P. rudis* and *P. nobilis* , respectively), reflecting both the limited representation of mollusc proteins in the databases and the expected high number of lineage‐specific gene content (Sun et al. 2024; Li et al. 2025).

The median protein length was approximately 290 amino acids for both species, with third quartile (Q3) values of 493 amino acids for *P. nobilis* and 536 amino acids for *P. rudis* (Figure S4). Comparison of the top 20 Gene Ontology (GO) terms across the three main categories (Biological Process, Molecular Function, and Cellular Component) revealed that, overall, both species have a similar number of annotated proteins, suggesting comparable functional profiles (Figure S5). Overall, the observed patterns point to strong structural similarity and functional conservation between the two species' predicted proteomes, regardless of their ecological or physiological differences. Notably, the greater abundance of DNA‐integration and replication genes in *P. nobilis* relative to *P. rudis* aligns with its elevated proportion of transposable elements.

### 3.2 Genome‐Wide Conservation and Orthology

Reciprocal BLAST searches between predicted coding DNA sequences (CDS) of *P. nobilis* and *P. rudis* identified 23,704 orthologous pairs, a number substantially lower than the total number of predicted CDS in each species but within the range expected for bivalve genomes (Dalquen and Dessimoz 2013). This discrepancy likely reflects the presence of paralogs arising from expanded gene families, incomplete or erroneous gene models, transposable element–derived open reading frames, as well as genuine lineage‐specific or fast‐evolving genes. Such sequences are unlikely to generate high‐confidence reciprocal best hits and are therefore excluded from RBH‐based ortholog detection.

A high degree of sequence conservation was observed among 20,300 filtered orthologs, with more than 75% displaying greater than 92% amino acid identity across their alignment lengths. Consistently, over 95% of orthologous gene pairs exhibited dN/dS ratios below 1, indicative of pervasive purifying selection and suggesting that the majority of protein‐coding genes are functionally conserved between the two species (Figure S6). Together, these results confirm the close evolutionary relationship between *P. nobilis* and *P. rudis* and indicate limited divergence at the amino acid level.

Whole‐genome alignments performed using Minimap2 with the ‐x asm20 preset (designed for up to 20% divergence) yielded a mean nucleotide identity of 86.5%. In contrast, alignment of coding sequences using the more stringent asm5 preset resulted in a mean identity of 97.8%. The high conservation of coding regions is consistent with expectations for congeneric species. However, because the asm20 preset primarily captures locally alignable and syntenic regions, highly divergent, rearranged, or repetitive genomic regions are likely underrepresented. Consequently, the observed 86.5% identity reflects only the fraction of the genome that could be reliably aligned, and overall genome‐wide divergence may be higher (Figure S7). These patterns are consistent with previous genome‐wide comparisons reported for species within the genus *Crassostrea* (Qi et al. 2023).

### 3.3 Transposable Elements and Repeat Landscape

Consistent with observations in other bivalve species, transposable elements (TEs) constitute a substantial proportion of both genomes analyzed. However, the majority of these elements could not be classified into known repeat families, a pattern frequently observed in molluscan genomes and likely reflecting the lineage‐specific nature or divergence of many TEs in these taxa (Zhang et al. 2012; Murgarella et al. 2016; Du et al. 2017; Martelossi et al. 2023).

Interestingly, the abundance of SINEs exceeded that of LINEs (including Penelope elements) in both species, contrary to the typical repeat composition observed in molluscs (Martelossi et al. 2023), where LINEs usually dominate. This unusual pattern may reflect a genus‐specific feature of RE composition in *Pinna*, possibly linked to the amplification of SINEs or the reduced retention of LINEs. However, an alternative (or complementary) explanation is that LINEs may be underrepresented due to their propensity for truncation, which complicates their identification and classification during repeat annotation.

The total abundance of annotated REs was broadly similar between the two *Pinna* species. Nonetheless, SINEs were slightly more abundant in the *P. rudis* genome, while LINEs were nearly 50% more abundant in *P. nobilis* (Table 2). This difference was largely driven by the overrepresentation of specific LINE subfamilies, particularly L2/CR1/Rex and RTE/Bov‐B, in *P. nobilis* . Such contrasting patterns of RE abundance may reflect distinct evolutionary trajectories, potentially shaped by differences in population history (e.g., bottlenecks), TE activity dynamics, or species‐specific environmental pressures. Given the role of transposable elements in genome architecture, gene regulation, and responses to environmental stress (Martelossi et al. 2023; Thomas‐Bulle et al. 2018; Doolittle 2022), these differences in repeat composition may have important implications for species resilience and adaptive potential, and thus warrant further investigation.

### 3.4 Gene Expansion in *Pinna nobilis* and *Pinna rudis*

Using a phylogeny‐based birth–death model of gene family evolution in bivalves, we identified likely trends of gene expansion in *P. nobilis* and *P. rudis* affecting multiple functional categories (Table 3). Both Pinna species displayed expansions in several gene families associated with cellular structure, stress response, and signaling, indicating a shared pattern of gene family diversification. Notably, genes annotated as nephrin−/contactin−/hemicentin‐like, belonging to the immunoglobulin superfamily, were expanded in both species. These genes are broadly associated with cell–cell adhesion and extracellular matrix organization, suggesting conserved enrichment of adhesion‐related molecular components. Expansions were also observed in multiple transposable element–related gene families, including PiggyBac‐ and Tigger‐derived proteins and other integrase‐associated domains, indicating an increased representation of mobile element–associated sequences in both genomes, consistent with the high proportion of repeat elements in both genomes. In addition, genes involved in membrane composition and lipid or glycan processing, such as GM2 ganglioside activator‐like proteins and lipid‐modifying enzymes, were expanded, highlighting shared diversification in pathways related to membrane‐associated processes.

Despite these similarities, differences in the functional composition of expanded gene families were observed between species. In *P. nobilis* , expanded families were enriched in genes encoding neurotransmission‐ and signaling‐related proteins, including acetylcholine receptor subunits, P2X purinoreceptors, and calcium‐binding proteins, as well as stress‐associated proteins such as serum amyloid A‐like and heat shock proteins. This species also showed a higher number of expansions involving retrovirus‐related and transposase‐ or integrase‐derived proteins. In contrast, *P. rudis* exhibited expansions predominantly in enzymatic gene families, including sulfotransferases, retinol dehydrogenases, and galactose sulfotransferases, as well as in genes related to lipid and glycan metabolism and cell‐surface or extracellular matrix–associated proteins.

Overall, while both species share expansions in gene families linked to structural organization, membrane‐associated functions, and mobile genetic elements, the distribution of expanded families differs in functional composition. *P. nobilis* shows a relative enrichment of signaling‐ and stress‐associated gene families, whereas *P. rudis* displays a higher representation of metabolic and surface‐associated functions. These patterns describe divergent trajectories of gene family expansion between the two species, while the majority of gene families remain conserved across their genomes. As this analysis focuses on detectable expansion events, additional lineage‐specific differences may exist that were not captured here.

Interestingly, several of the expanded gene families identified here have been previously associated with cellular processes involved in stress responses, signaling, and membrane‐associated functions in invertebrates, including pathways that are often discussed in the context of host–pathogen interactions (Zhu, Hu, et al. 2018; Vogel and Hedgecock 2001; Coyne 2011; Dubyak 2012). While these observations do not constitute direct evidence of adaptation to pathogens, they indicate that gene family expansions in these functional categories may be relevant to differences in cellular responses to environmental and biological challenges. Consequently, variation in the composition of expanded gene families between *P. nobilis* and *P. rudis* could be consistent with a partial contribution to their contrasting susceptibility to intracellular infections, a hypothesis that warrants targeted functional and experimental investigation.

### 3.5 Comparison of Immune‐, Stress‐, and Detoxification‐Related Gene Families in *Pinna nobilis* and *Pinna rudis*

Comparative analysis of functional annotations revealed broadly similar gene family repertoires between *P. nobilis* and *P. rudis* , with differences mainly affecting detoxification and stress response categories (Table 4). Many proteins contained multiple immunoglobulin domains, often annotated as hemicentin‐like, which are highly abundant in bivalve genomes and often associated with repetitive elements. Yet their precise biological functions remain largely unknown and thus these annotations should be interpreted with caution. Both species exhibited expanded gene families associated with putative innate immune pattern‐recognition receptors (PRRs), including C1q‐domain‐containing proteins, fibrinogen C‐terminal domain‐containing proteins, and C‐type lectins. Detoxification functions were well represented, with more than 140 cytochrome P450 annotations, and the stress response pathways were characterized by high numbers of heat shock proteins, particularly Hsp70 and Hsp40 (DnaJ). Notably, *P. nobilis* showed slightly higher numbers of cytochrome P450 and Hsp70 annotations compared to *P. rudis* , suggesting potential differences in detoxification‐ and stress‐associated gene families.

A similar pattern emerged from the GO enrichment analysis, which indicated broadly comparable numbers of genes annotated to immune‐, stress‐, and detoxification‐related biological processes in both species (Table S2). The GO term “inflammatory response” (GO:0006954) was more frequently assigned in *P. rudis* (70 annotations) than in *P. nobilis* (59 annotations), and a substantial proportion of these annotations corresponded to rhodopsin‐like G protein–coupled receptors (GPCRs), including sequences with similarity to vertebrate prostanoid receptors. In bivalves, GPCRs represent a large and highly diversified gene family whose evolutionary trajectories and functional roles are only partially understood, and their annotation to immune‐related GO terms largely reflects homology‐based inference. Consequently, while GPCRs with immune‐associated annotations may participate in cellular signaling pathways relevant to host–environment interactions (Box et al. 2020; Schmid and Brüne 2021; Lattos et al. 2021; Sun and Ye 2012; Zhu, Yang, et al. 2018), their specific involvement in inflammatory or immune processes in bivalves remains unresolved, and functional interpretations based solely on sequence similarity to vertebrate receptors should be treated with caution.

### 3.6 An Update Description of the Toll‐Like Receptor Repertoires

**FIGURE 2:**
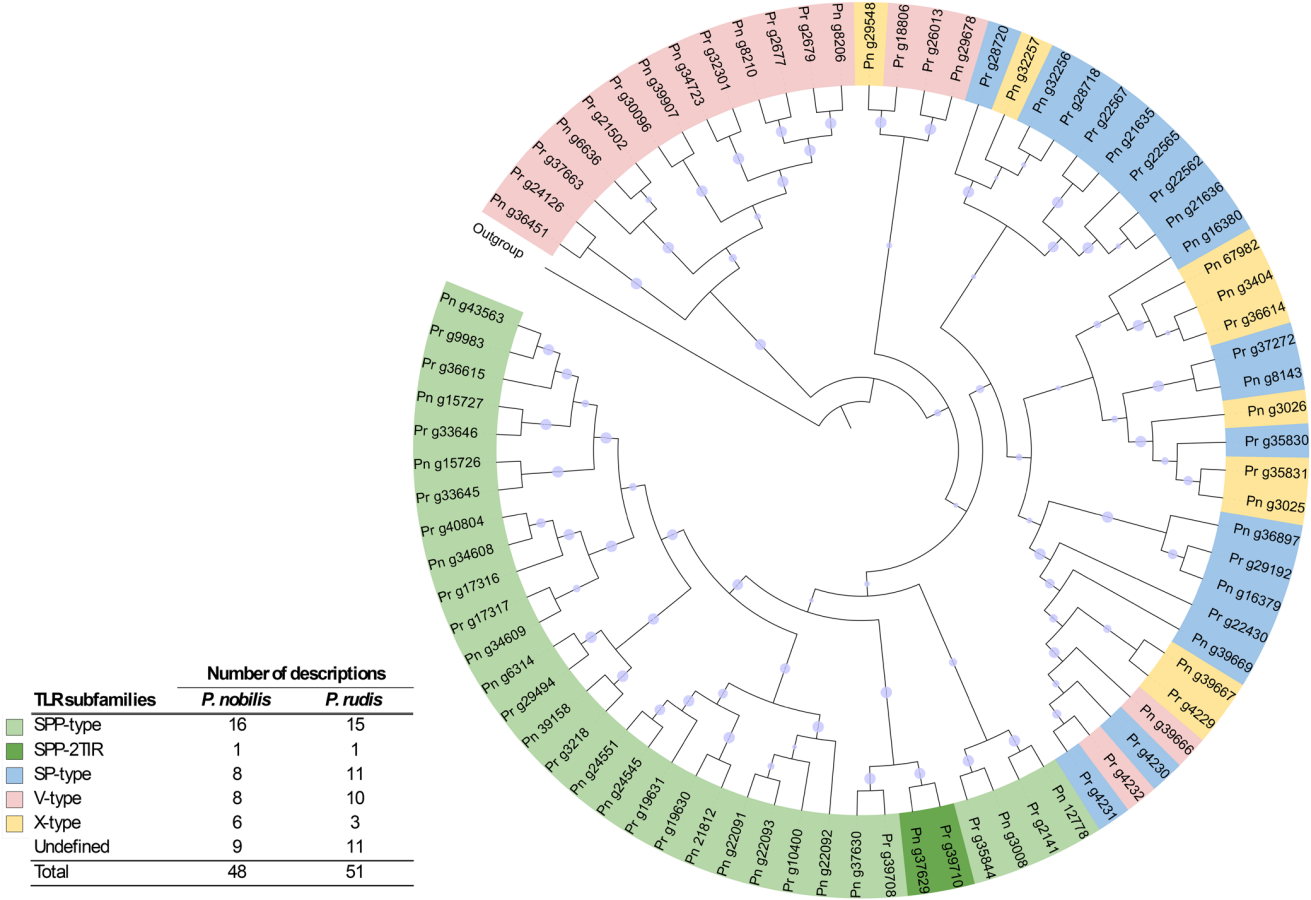
Phylogenetic relationships among predicted TLR coding sequences in *Pinna rudis* and *P. nobilis* . The tree was constructed based on the amino acid sequences of the conserved TIR domains and rooted using the TIR domain of a predicted TLR from the sponge *Amphimedon queenslandica* (XP_003383414.2). TLR subfamilies, classified according to the nomenclature proposed for bivalves (Gerdol et al. 2017), are indicated by different colors; Undefined: TLR are not considered in the analysis. “Pn” and “Pr” denote sequences from *Pinna nobilis* and *Pinna rudis* , respectively. Labels at the ends of branches correspond to the contig names within the predicted coding sequences of both genomes.

The two species exhibit a similar repertoire of predicted Toll‐like Receptors (TLRs), with numbers consistent with those reported in other bivalve genomes. All TLR subfamilies defined by the classification of Gerdol et al. (Gerdol et al. 2017; Saco et al. 2023). were detected, with the SPP‐type subfamily representing approximately 40% of all classified TLRs. Among the identified TLRs, protein lengths ranged from 370 to 1252 amino acids in *P. nobilis* , and from 201 to 1343 amino acids in *P. rudis* . The phylogenetic analysis based on the conserved TIR domain supports the classification of TLRs into distinct subfamilies, i.e., SPP‐type, V‐type, and a mixed group including SP‐, V‐, and X‐types, each forming well‐supported clades (Figure 2). The analysis also indicates that the majority of TLRs are conserved between the two species. However, 6 TLRs in *P. nobilis* and 9 in *P. rudis* lack identifiable orthologs. This discrepancy may reflect genome assembly fragmentation or true presence/absence variation among species and individuals (Gerdol et al. 2020). For instance, four TLRs previously reported in *P. nobilis* were not detected in the current *P. nobilis* assembly.

**TABLE 1:** BUSCO assessment of genome assembly and gene model completeness for *P. nobilis* and *P. rudis* (mollusca_odb12).

BUSCO	*Pinna rudis*		*Pinna nobilis*		
	New assembly	Gene model	New assembly	Gene model	Previous assembly
Complete (%)	96.5	92.9	96.5	93.1	30.1
Single (%)	94.8	91.3	95.4	91.9	29.7
Duplicated (%)	1.7	1.6	1.1	1.2	0.4
Fragmented (%)	1.7	4.6	2.5	5.2	42.1
Missing (%)	1.8	2.6	1.1	1.7	27.8

**TABLE 2:** Statistics of whole‐genome sequence assemblies of *Pinna nobilis* and *P. rudis* .

	*Pinna nobilis*	*Pinna rudis*
**Accession number**	GCA_054095635.1	GCA_054095625.1
Total of sequenced length (nt)	36,696,267,450	17,886,916,570
No. of reads > 1000 nt	8,527,691	2,907,775
No. of reads > 5000 nt	2,227,609	1,556,964
No. of reads > 10,000 nt	347,627	337,924
**Primary assembly**		
Length (nt)	1,044,252,029	1,413,358,278
No. of contigs	23,807	22,820
**Final assembly (all contigs)**		
Length (nt), N included	812,778,662	761,551,605
N's per 100 Kb	2.85	0.58
Largest contig (nt)	3,175,472	4,056,875
No. of contigs	4267	3422
Final assembly (contigs longer than 30 Kb)	794,795,419	727,132,473
N's per 100 Kb	2.83	0.58
No. of contigs (> 30,000 nt)	2948	1869
N50 (nt)/L50	439,336/548	657,993/332
N90 (nt)/L90	130,318/1811	180,600/1142
**Repeat elements: bases masked (% of assembly)**	359,221,427 (45.2)	318,256,528 (42.84)
Retro‐elements (No/% of sequences)	294,924 (7.1)	304,301 (7.88)
SINE	246,118 (4.94)	265,856 (6.05)
Penelope	107,702 (2.7)	98,784 (2.47)
LINE	34,090 (1.29)	22,450 (1.03)
LTR	14,716 (0.88)	15,995 (0.81)
DNA transposons (No/% of sequence)	108,597 (2.52)	105,186 (2.23)
Simple repeats (No/% of sequence)	205,874 (1.55)	202,822 (1.54)
Unclassified (No/% of sequence)	1,204,962 (30.93)	1,106,968 (28.26)

**TABLE 3:** Expanded genes in *Pinna nobilis* and *P. rudis* . Trends in gene family expansion along the Pinna lineages inferred from the root of the reconstructed bivalve phylogeny using a phylogeny‐based birth–death model implemented in CAFE. Expanded gene families are grouped into functional categories for clarity.

Annotation	*P. nobilis*	*P. rudis*
**Calcium/Signaling Proteins**		
Calmodulin	7	
**DNA/RNA Binding/Nucleases/Regulatory**		
THAP domain‐containing protein 1	3	
Zinc finger protein 862‐like	3	
Uncharacterized protein KIAA1958‐like / zinc finger MYM‐type protein 3‐like		8
**Enzymes/Catalytic**		
Carbohydrate sulfotransferase 10‐like		5
Polyprenol dehydrogenase‐like isoform X1 / retinol dehydrogenase 12‐like		4
Galactose‐3‐O‐sulfotransferase 2‐like / galactosylceramide sulfotransferase‐like isoform X2		4
**Lipid/Glycan Binding/Metabolism**		
TLC domain‐containing protein 4‐like	4	
Ganglioside GM2 activator‐like		6
**Neurotransmission/Receptors**		
Neuronal acetylcholine receptor subunit alpha	18	
P2 × purinoreceptor 7‐like	15	
PRRT1 (prolin‐rich transmembran protein 1‐like)	4	
Acetylcholine receptor subunit beta‐like 1	3	
**Stress/Chaperone Proteins**		
Serum amyloid A‐like protein / Major acute phase reactant	3	
heat shock 70 kDa protein 12B‐like	3	
**Structural/ECM**		
Fibropellin‐1‐like protein / fibrillin‐2	14	4
Ig–FN3 with TM domaion adhesion proteins annotated as nephrin/contactin/hemicentin	10	15
Short‐chain collagen C4	5	
**Transporters**		
Organic cation transporter	5	2
**Transposable Elements/Integrases**		
RT et integrase domain (DDE)	7	
Transposase PiggyBac/piggyBac transposable element–derived protein 4‐like	5	11
Integrase/recombinase xerD homolog	4	
Tigger transposable element–derived protein 6‐like		8

**TABLE 4:** Comparative counts of defensome‐related gene families involved in transport, innate immunity, antioxidant defense, detoxification, and stress response in *Pinna nobilis* and *P. rudis* .

Gene	*P. nobilis*	*P. rudis*
**Transporters**		
ATP binding cassette (family A)	1	1
ATP binding cassette (family B)	5	6
ATP binding cassette (family C)	6	5
ATP binding cassette (family D)	3	2
ATP binding cassette (family E)	1	1
ATP binding cassette (family F)	5	6
ATP binding cassette (family G)	9	9
**Innate immune PRR**		
Peptidoglycan recognition protein	6	4
Glucan binding protein	3	3
C‐type lectins	18	13
C‐type mannose receptor	7	7
Collectin‐like	7	10
Galectin recognition domain	8	8
LysM	2	2
Fibrinogen C‐terminal domain	38	36
C1q total	115	118
C1q‐TNF	30	30
C1q‐like protein	47	44
**Antioxidants**		
Catalase	1	1
Superoxide dismutase	6	7
Glutathione peroxidase	10	13
Glutathione reductase	1	1
**Detoxification**		
Cytochrome P450	156	141
**Stress response/molecular chaperones**		
HSP90	1	1
HSP70	133	103
HSP68	4	5
HSP60 (mitochondrial)	1	1
HSP40 (DnaJ)	40	42
HSP10	1	1

The percent identity of TLR coding sequences among the 33 orthologs ranged from 80% to 99% (mean = 94.5%, SD = 3,56%). Corresponding dN/dS ratios calculated over the full‐length coding regions varied from 0.11 to 0.93, with 90% of ortholog pairs exhibiting a dN/dS ratio below 0.66 (mean = 0.38, SD = 0.17). The observed low dN/dS ratios are consistent with strong purifying selection acting to conserve protein function across species. This aligns with the established role of TLRs as key components of the innate immune system, where functional integrity is typically maintained. However, the presence of ortholog pairs with relatively higher dN/dS ratios (0.43–0.93) suggests localized relaxation of constraint or episodes of adaptive divergence in certain lineages or domains. Notably, these elevated dN/dS values were not restricted to a specific TLR subfamily but were distributed across all structural classes, indicating that potential adaptive changes may occur broadly across the TLR repertoire, rather than being confined to a single evolutionary lineage or structural group. These findings support a model in which TLRs are generally conserved but retain pockets of evolutionary flexibility, potentially enabling responsiveness to lineage‐specific pathogenic environments.

## 4 Conclusion

In this study, we present new genomic resources that are critical for the conservation of *P. nobilis* and for understanding the population dynamics of *P. rudis* across the Mediterranean basin. Although the assemblies do not yet reach chromosome‐level resolution, their completeness and the number of annotated proteins are comparable to those of recently published mollusc genomes. Despite subtle differences in genome size, repeat content, and gene family expansions, the two species exhibit high similarity at the putative transcriptomic and proteomic levels, reflecting their close evolutionary relationship and supporting the possibility of interspecific gene flow. Comparative analyses reveal largely conserved functional gene repertoires between both species, together with notable divergence in detoxification pathways, stress‐response genes, and inflammatory response annotations. In addition, the observed trends in gene family expansion open new research perspectives aimed at linking these expansions to species‐specific responses to pathogen exposure. Collectively, these results highlight both shared evolutionary constraints and adaptive differences between the two species. Finally, these genome assemblies constitute essential resources for future research and provide valuable tools to support the conservation and management of these ecologically important and increasingly imperiled bivalves. Under the current high biotic selection pressures experienced by Mediterranean Pinna populations, the comprehensive gene annotations presented here offer a strong foundation for comparative genomic studies aimed at identifying loci potentially including introgressed variants associated with resistance to mass mortality events.

## Author Contributions

**Stéphane Coupé:** conceptualization (lead), data curation (lead), formal analysis (lead), funding acquisition (supporting), investigation (lead), methodology (lead), project administration (lead), resources (equal), software (lead), supervision (lead), validation (equal), visualization (lead), writing – original draft (lead), writing – review and editing (equal). **Mathieu Foulquié:** funding acquisition (equal), investigation (equal), resources (lead), validation (equal), writing – review and editing (equal). **Maite Vázquez Luis:** funding acquisition (equal), resources (equal), writing – review and editing (equal). **Elvira Alvarez Perez:** funding acquisition (equal), resources (equal), writing – review and editing (equal). **Jean‐Marc Prévot:** software (equal), writing – review and editing (equal). **Nardo Vicente:** writing – review and editing (equal). **Robert Bunet:** conceptualization (equal), funding acquisition (equal), investigation (equal), project administration (equal), resources (equal), writing – review and editing (equal).

## Funding

This work was supported by the University of Toulon related to the PINORES project: “The study of adaptive changes of pen shell (*Pinna nobilis*) to the pathogen *Haplosporidium pinnae*,” the University Institute of Technology of the University of Toulon under the grant “CARTT,” and by the European Union's LIFE programs through the project LIFE PINNARCA (LIFE20‐NAT/ES/001265) and LIFE24‐NAT‐ES‐PINNACARE/101216239.

## Conflicts of Interest

The authors declare no conflicts of interest.

## Supporting information


**Table S1:** BUSCO completeness and assembly statistics of *Pinna nobilis* and *P. rudis* genome assemblies under different contig subsampling levels. BUSCO completeness was assessed using the metaeuk_odb10 dataset, with S, single‐copy; D, duplicated; F, fragmented, and M, missing BUSCOs. Assembly statistics include total assembly size (Mb), number of contigs, and contiguity metrics (L_50_ and L_50_).
**Table S2:** GO biological processes associated with immune system, stress response and detoxification pathways in Pinna nobilis and P. rudis.
**Figure S1:** Pairwise percent identity of syntenic orthologs between Crassostrea gigas and C. angulata.
**Figure S2:** Cumulative genome length plots.
**Figure S3:** Top 25 most frequent species in the annotated genomes.
**Figure S4:** Protein length distributions.
**Figure S5:** Top 20 Gene Ontology (GO) terms by occurrence for the three GO categories: Biological Process, Cellular Component, and Molecular Function.
**Figure S6:** Pairwise percent identity and dN/dS ratios of orthologous proteins between Pinna nobilis and P. rudis.
**Figure S7:** Alignment identity distribution.

## Data Availability

Genome assemblies for *P. rudis* and *P. nobilis* have been deposited in GenBank under accession numbers GCA_054095625.1 and GCA_054095635.1, respectively. Raw sequencing reads are available from the NCBI Sequence Read Archive under BioProject accession numbers PRJNA1346463 and PRJNA1346468. Annotation data and predicted TLR proteins are available at the Dryad address: https://doi.org/10.5061/dryad.0rxwdbsf2.
