# Supplementary material for: Comparative Genomics of Pinna rudis and Pinna nobilis Reveals Conserved and Divergent Features of the Bivalve Defensome
Source: Ecol Evol. 2026 Mar 25;16(3):e73267. doi: 10.1002/ece3.73267 (PMC13093298; doi:10.1002/ece3.73267)
Supplement: Supplementary file 1 — Table S1: BUSCO completeness and assembly statistics of Pinna nobilis and P. rudis genome assemblies under different contig subsampling levels. BUSCO completeness was assessed using the metaeuk_odb10 dataset, with S, single‐copy; D, duplicated; F, fragmented, and M, missing BUSCOs. Assembly statistics include total assembly size (Mb), number of contigs, and contiguity metrics (L50 and L50). Table S2: GO biological processes associated with immune system, stress response and detoxification pathways in Pinna nobilis and P. rudis. Figure S1: Pairwise percent identity of syntenic orthologs between Crassostrea gigas and C. angulata. Figure S2: Cumulative genome length plots. Figure S3: Top 25 most frequent species in the annotated genomes. Figure S4: Protein length distributions. Figure S5: Top 20 Gene Ontology (GO) terms by occurrence for the three GO categories: Biological Process, Cellular Component, and Molecular Function. Figure S6: Pairwise percent identity and dN/dS ratios of orthologous proteins between Pinna nobilis and P. rudis. Figure S7: Alignment identity distribution. [file ECE3-16-e73267-s001.docx]

*Comparative genomics of Pinna rudis and Pinna nobilis reveals conserved and divergent features of the bivalve defensome*

**Stéphane Coupé^1^, Mathieu Foulquié^1,2^, Maite Vázquez Luis^3^, Elvira Alvarez Perez^3^, Jean-Marc Prévot^4^, Nardo Vicente^2,5^ & Robert Bunet^2^.**

1. Université de Toulon, Aix Marseille Univ, CNRS, IRD, MIO, Marseille, France.

2. Institut océanographique Paul Ricard, Ile des Embiez, Var, France.

3. Instituto Español de Oceanografía (IEO, CSIC), Centro Oceanográfico de Baleares. Muelle de poniente s/n, 07015. Palma de Mallorca, Spain.

4. Département informatique, Université de Toulon, Var, France.

5. Institut Méditerranéen de Biodiversité et Ecologie marine et continentale (IMBE), Aix-Marseille Université, CNRS, IRD, Avignon Université, France

**^*^**To whom correspondence should be addressed. Email: [stephane.coupe@mio.osupytheas.fr](mailto:stephane.coupe@mio.osupytheas.fr)

**Supporting information**

**Table 1. BUSCO completeness and assembly statistics of *Pinna nobilis* and *P. rudis* genome assemblies under different contig subsampling levels.** BUSCO completeness was assessed using the metaeuk_odb10 dataset, with S = single-copy, D = duplicated, F = fragmented, and M = missing BUSCOs. Assembly statistics include total assembly size (Mb), number of contigs, and contiguity metrics (L₅₀ and L₉₀).

**Table 2. GO biological processes associated with immune system, stress response and detoxification pathways in *Pinna nobilis* and *P. rudis*.**


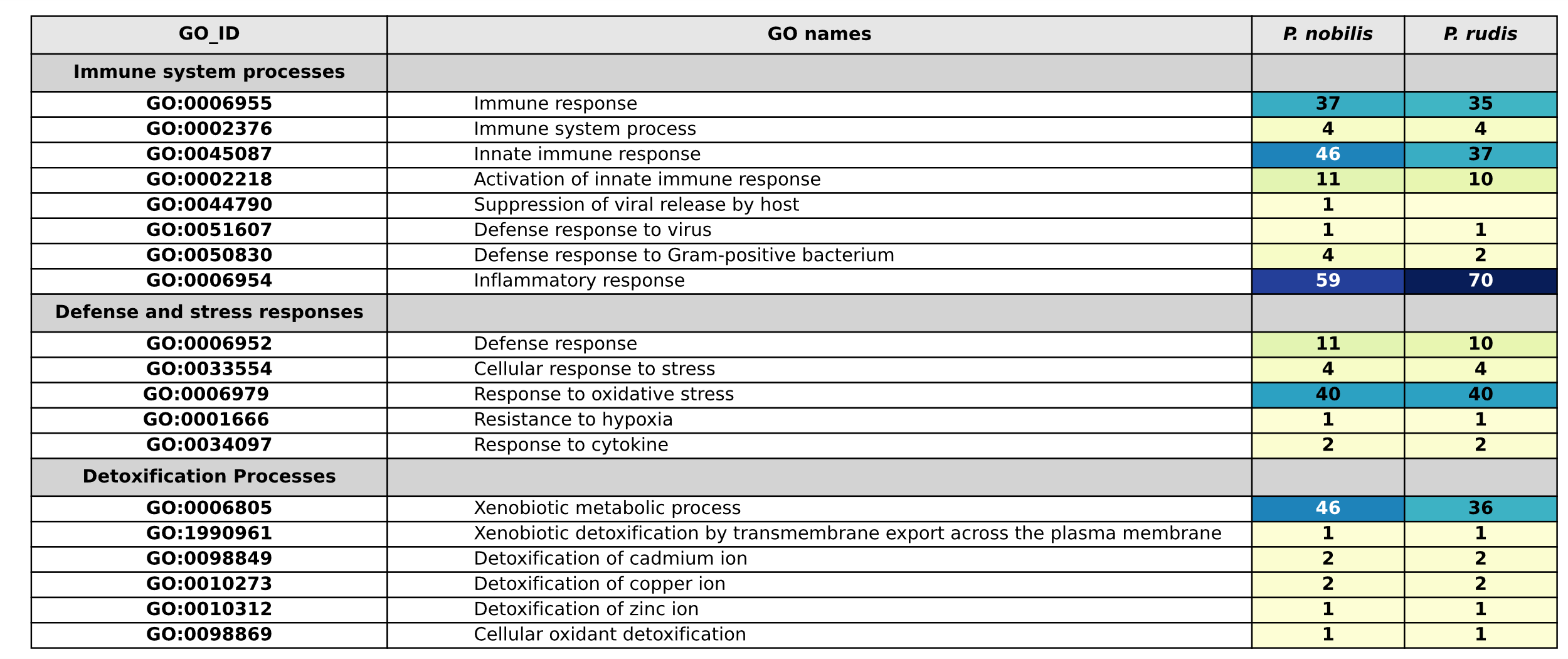


**Figure 1. Pairwise percent identity of syntenic orthologs between *Crassostrea gigas* and *C. angulata*.**

Using MCScanX, 34,346 collinear genes were identified out of 55,883 genes (61.46%). A total of 17,713 syntenic gene pairs were detected using default parameters (minimum of five collinear genes per block), of which 7,855 pairs were supported by BLAST matches.

**
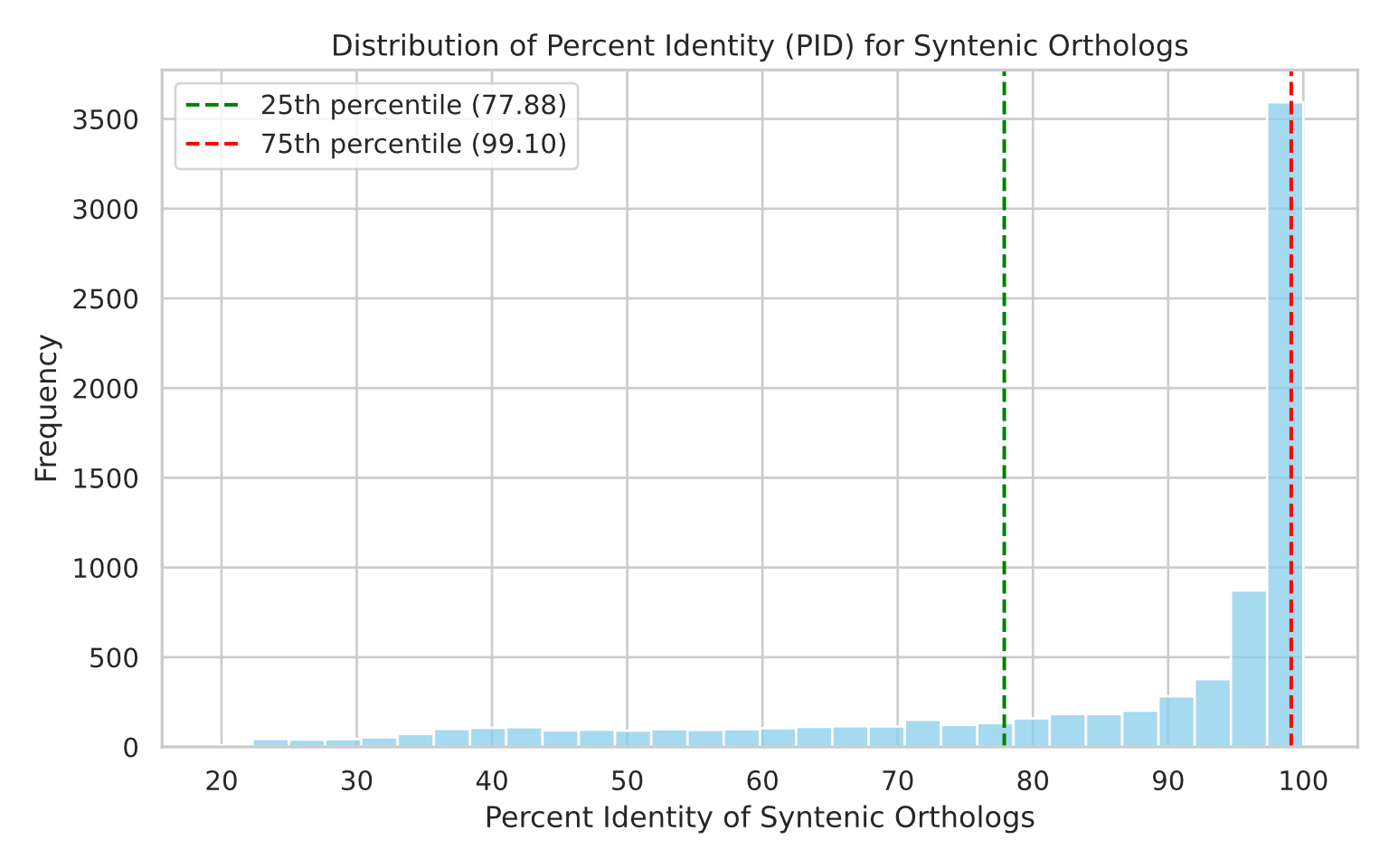
**

**Figure 2. Cumulative genome length plots.**

**Figure 3. Top 25 most frequent species in the annotated genomes.**

**Figure 4. Protein length distributions.**

**Figure 5. Top 20 Gene Ontology (GO) terms by occurrence for the three GO categories: Biological Process, Cellular Component, and Molecular Function.**

**Figure 6. Pairwise percent identity and dN/dS ratios of orthologous proteins between *Pinna nobilis* and *P. rudis*.**


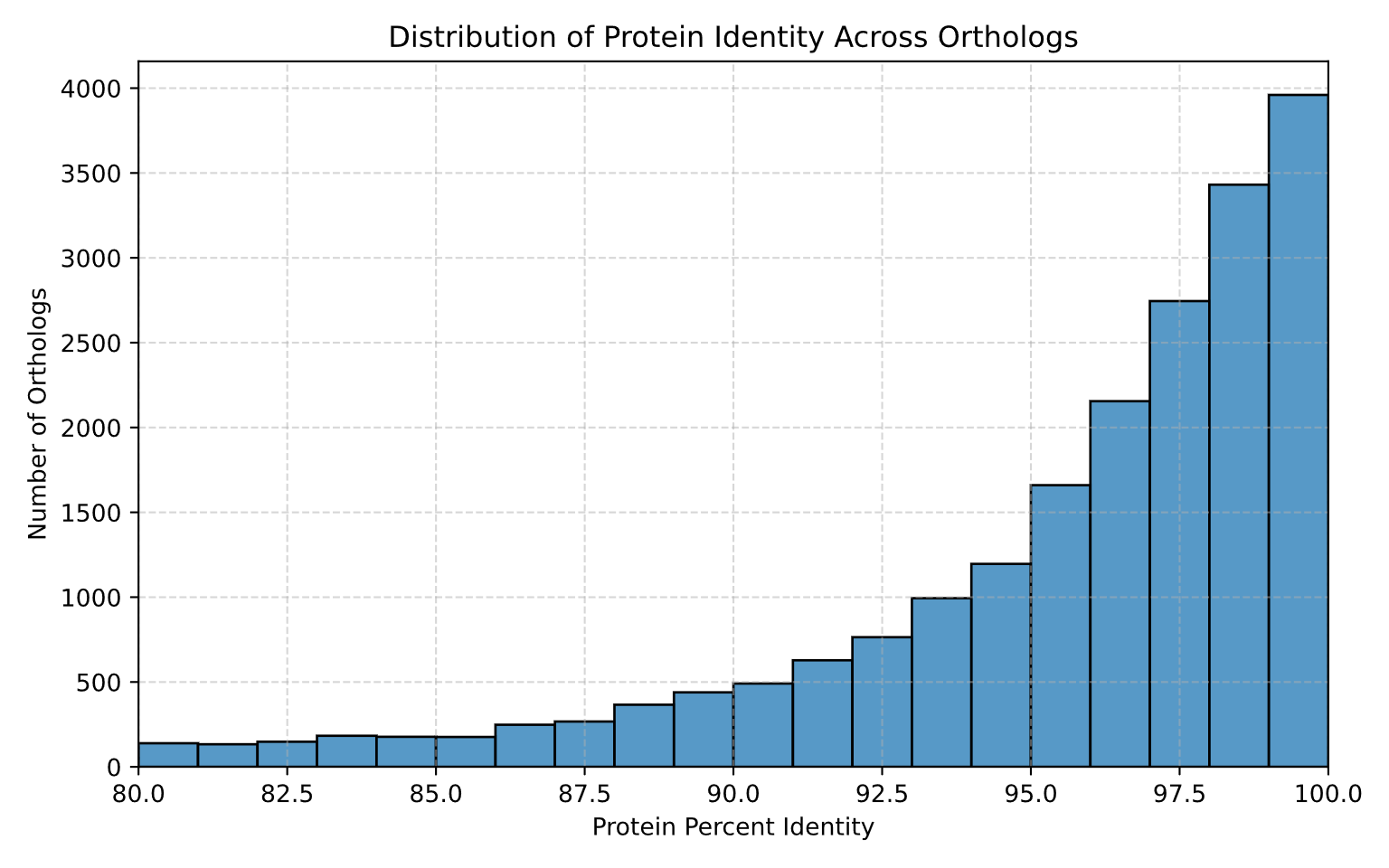


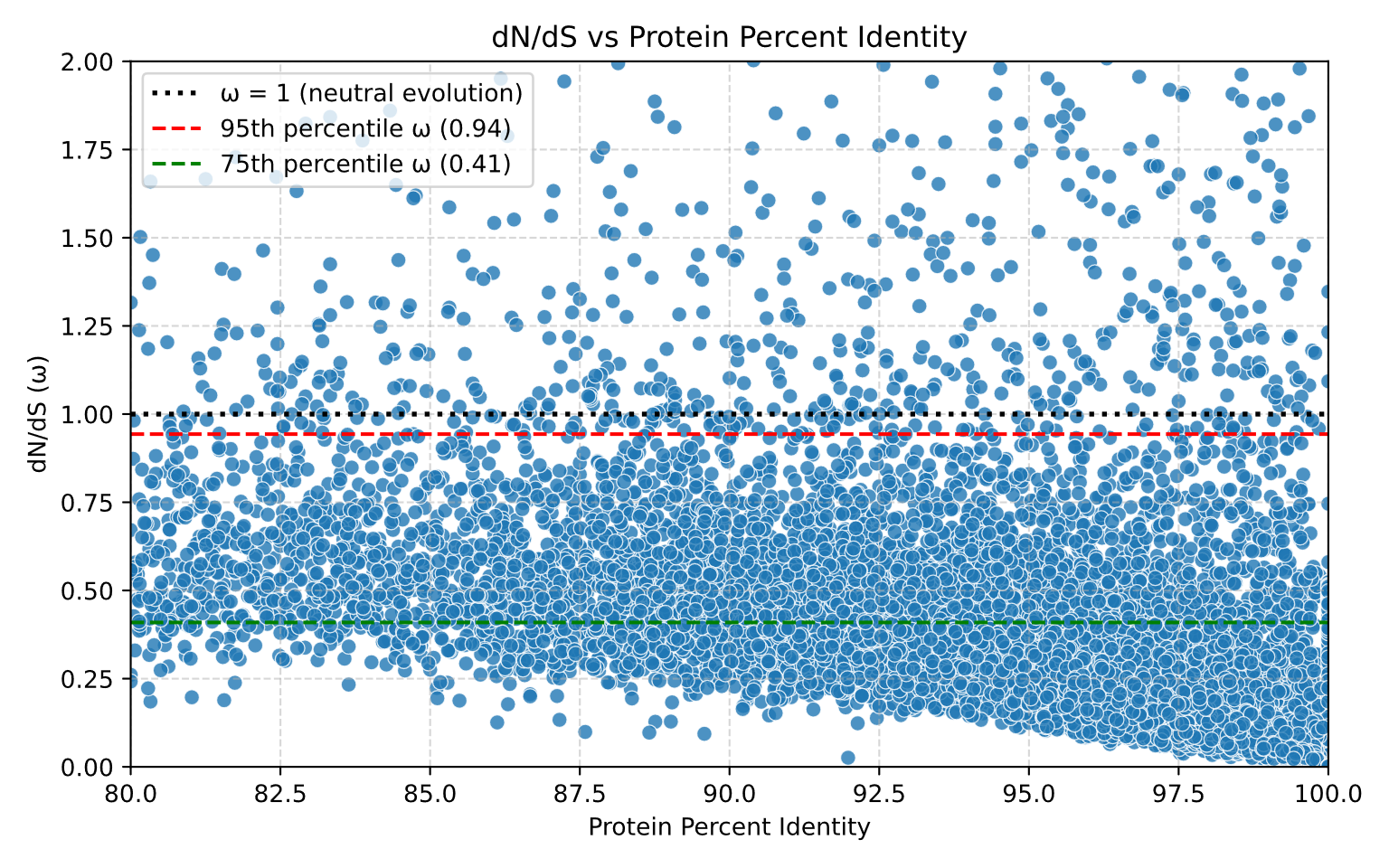


**Figure 7. Alignment identity distribution.**
